# Metabolic Derangement in Pediatric Patient with Obesity: The Role of Ketogenic Diet as Therapeutic Tool

**DOI:** 10.3390/nu13082805

**Published:** 2021-08-16

**Authors:** Valeria Calcaterra, Elvira Verduci, Martina Chiara Pascuzzi, Vittoria Carlotta Magenes, Giulia Fiore, Elisabetta Di Profio, Elisavietta Tenuta, Alessandra Bosetti, Carolina Federica Todisco, Enza D’Auria, Gianvincenzo Zuccotti

**Affiliations:** 1 Pediatric and Adolescent Unit, Department of Internal Medicine, University of Pavia, 27100 Pavia, Italy; valeria.calcaterra@unipv.it (V.C.); elisavietta.tenuta01@universitadipavia.it (E.T.); 2 Pediatric Department, “Vittore Buzzi” Children’s Hospital, 20154 Milan, Italy; martina.pascuzzi@unimi.it (M.C.P.); vittoria.magenes@unimi.it (V.C.M.); giulia.fiore2@studenti.unimi.it (G.F.); elisabetta.diprofio@unimi.it (E.D.P.); alessandra.bosetti@asst-fbf-sacco.it (A.B.); carolina.todisco@unimi.it (C.F.T.); enza.dauria@unimi.it (E.D.); 3 Department of Health Sciences, University of Milano, 20142 Milano, Italy; 4 Department of Biomedical and Clinical Science “L. Sacco”, University of Milan, 20157 Milan, Italy

**Keywords:** obesity, ketogenic diet, insulin resistance, lipids, diabetes

## Abstract

Obesity is defined as a condition characterized by an excessive fat accumulation that has negative health consequences. Pediatric obesity is associated with an increased risk for many diseases, including impaired glycemic and lipidic control that may lead to the development of chronic, and potentially disabling, pathologies, such as type 2 diabetes mellitus (T2DM) and cardiovascular events, in adult life. The therapeutic strategy initially starts with interventions that are aimed at changing lifestyle and eating behavior, to prevent, manage, and potentially reverse metabolic disorders. Recently, the ketogenic diet (KD) has been proposed as a promising dietary intervention for the treatment of metabolic and cardiovascular risk factors related to obesity in adults, and a possible beneficial role has also been proposed in children. KD is very low in carbohydrate, high in fat, and moderate to high in protein that may have the potential to promote weight loss and improve lipidic derangement, glycemic control, and insulin sensitivity. In this review, we present metabolic disorders on glycemic and lipidic control in children and adolescents with obesity and indication of KD in pediatrics, discussing the role of KD as a therapeutic tool for metabolic derangement. The results of this review may suggest the validity of KD and the need to further research its potential to address metabolic risk factors in pediatric obesity.

## 1. Introduction

Obesity is a complex, many-faceted status that exposes the pediatric patients to a wide spectrum of inflammatory, metabolic, and endocrine dysfunctions, which influence and enhance each other through biochemical and molecular interactions [1]. Pediatric obesity exposes the affected subjects to a higher risk of short- and long-term complications, such as type 2 diabetes (T2DM), dyslipidemia, nonalcoholic fatty liver disease (NAFLD), obstructive sleep apnea (OSA), asthma, polycystic ovary syndrome (PCOS), musculoskeletal comorbidities [2], and adolescents with obesity are at increased risk of psychological disturbances [3,4,5].

Early metabolic disorders, including insulin resistance, prediabetes, and dyslipidemia, are metabolic disorders that are often detected in obese children and adolescents [6]. They have a particular relevance because of their strong association with the development of chronic, and potentially disabling, pathologies, such as type two diabetes mellitus (T2DM) and cardiovascular events, in adult life [7]. Considering the short- and long-term risk complications, pediatricians and specialists should be aware of the frequency and extent of obesity-related comorbidities, and address patients to periodic screening procedures [6]. It is essential to follow the children who are affected by obesity regularly and in a multidisciplinary team. The strategy initially starts with interventions aimed at changing lifestyle and dietary interventions, to prevent, manage, and potentially reverse metabolic disorders [1]. Recently, the ketogenic diet (KD) has been proposed has dietary intervention that may have the potential to promote weight loss, and improve lipidic derangement and glycemic control/insulin sensitivity in adults [8,9].

In pediatrics, a possible therapeutic role in treating chronic inflammatory disease in children has been reported [10].

The purpose of this review is to present metabolic disorders on glycemic and lipidic control in children and adolescents with obesity and indication of KD in pediatrics, discussing the biological plausibility of the mechanisms by which the KD reduces the metabolic derangement that is currently reported for adults. The results of this review could suggest the validity of KD and its potential to address metabolic risk factors in pediatric obesity. A novel non-pharmacological treatment may be useful as an alternative tool in managing children with metabolic derangement. A prompt identification and correction of these abnormalities is fundamental, to significantly decrease the likelihood of adverse events throughout life.

## 2. Methods

We performed a narrative review [11] according to the English literature in the past 15 years. M.C.P., V.C.M., G.F., E.D.P., E.T., C.F.T. independently identified the most relevant published studies including original papers, metanalysis, clinical trials, reviews. Case reports or series and letters were excluded. Adult and pediatric literature was considered. Regarding pathogenetic mechanism experimental studies were also included. Papers published up to May 2021 in each author’s field of expertise were searched with the following keywords (alone or in combination): obesity, adolescents, obesity-related complications, dyslipidemia, glycemic, glucose impairment, insulin resistance, prediabetes metabolic risk, metabolic syndrome, type 2 diabetes, ketogenic diet, ketogenic diet and metabolic syndrome, ketogenic diet and cardiovascular disease, very-low-carbohydrates ketogenic diet, very-low-carbohydrates ketogenic diet and diabetes or insulin resistance or polycystic ovary syndrome. The following electronic databases were searched: PubMed, Scopus, EMBASE and Web of Science.

## 3. Pediatric Obesity

### 3.1. Epidemiological Data

Obesity is defined as a condition characterized by an excessive fat accumulation that has negative health consequences, being a risk for many diseases, including a wide spectrum of metabolic and cardiovascular disorders [1]. The condition is extremely diffused worldwide, and represents a major public health problem in both childhood and adulthood [12,13].

Obesity has increased significantly in the last four decades, both in industrialized and developing countries, and it is of major relevance in the pediatric age. The global prevalence of overweight and obesity in males and females aged 5–19 has risen from 4% in 1975 to 18% in 2016 [14]. According to the World Health Organization report, in 2016, more than 340 million children and adolescents worldwide were in a condition of excess body weight. Consistent with the pooled analysis of 2416 population-based studies, performed by Non-Communicable Diseases (NCD) Risk Factor Collaboration (NCD-RisC), obesity has increased by 4.9% in females (from 0.7% in 1975 to 5.6% in 2016) and 6.9% in males (from 0.9% to 7.8%) [15].

Italy is one of the nations with the highest levels of excess weight in children; about one in four children have a weight greater than that expected for their age. The prevalence of overweight and obesity in 2019 was 20.4% and 9.4%, respectively, with the highest number of cases in the south. Obesity was slightly higher in males (9.9% vs. 8.8% in females) [16].

### 3.2. Risk Factors for Obesity

Essential obesity is a multifactorial and complex condition, whose fundamental basis is an unbalanced relationship between caloric intake and energy expenditure [17]. This mainly derives from unhealthy eating habits or an excess sedentary lifestyle, and typically results from both. A diet that is rich in highly processed foods, with frequent consumption of sweetened drinks, ready-to-eat snacks, and fast-food preparations, constitutes an important contribution to the development of childhood obesity [4]. On the other hand, nowadays the tendency to enroll children at a sport activity has been reduced, simultaneously, the time spent in sedentary activities, such as use of the television and video games, has increased [5]. The changes in lifestyles create an “obesogenic” environment, which contributes to explaining the high prevalence of the condition.

Other risk factors are involved, as follows: there is a strong genetic susceptibility, confirmed by the strong association between obesity in first-degree relatives and the risk for the child to become overweight. Moreover, twins tend to have similar BMI [18]. Genetics would therefore play a permissive role, interacting with environmental factors that promote obesity.

In addition to those already mentioned, the risk factors for obesity include the following: low socio-economic level, urban area of residence compared to rural, Hispanic and South Asian ethnicity, psychosocial and emotional factors (where food becomes a mean to suppress feelings and negative moods), intestinal bacterial flora composition (for example, the relationship between Firmicutes and Bacteroidetes), and a poor quality and duration of sleep [17]. Sleep may also be related to glycemic homeostasis and insulin sensitivity, as described in a cross-sectional observational study that was conducted by Koren and co-authors, between 62 obese adolescents. From the study emerged a significant correlation between sleep duration, HbA1c values, and glucose levels, with the oral glucose load test (OGTT), regardless of gender, degree of obesity, and pubertal stage [19]. Insufficient and excessive sleep was associated with short-term and long-term hyperglycemia in the obese study group. Decreased slow-wave sleep (phase N3) was associated with reduced insulin secretion [19]. 

Other conditions that are associated with a higher risk of excessive adiposity are intrauterine exposure to the mother’s excess adiposity, gestational diabetes, and small-for-gestational-age (SGA) newborns, who show a subsequent early recovery of growth [17]. During pregnancy, the differentiation of fetus hypothalamic hunger and satiety centers occurs, and the number of adipocytes increases. Accordingly, an overstimulation of these centers, during the intrauterine life, predispose to obesity. 

### 3.3. Diagnosis of Overweight and Obesity

The diagnosis of overweight and obesity in children up to two years of age is based on the weight-to-length ratio, using the WHO child growth standards reference curves for age and sex [6,20]. After two years of age, the diagnosis is made using the parameter body mass index (BMI), calculated by dividing the child’s weight, expressed in kilograms, by the square of their height, measured in meters (kg/m^2^) [6]. Even though BMI in pediatrics is calculated using the same formula as adult BMI, it is interpreted differently. In adults, BMI is interpreted using standard weight status categories. On the contrary, at pediatric age, the BMI calculated is compared with standard reference curves that are gender-, age-, and population-specific, because the amount of body fat changes with age, and the amount of body fat differs between girls and boys. The adoption of reference curves with normative BMI percentiles considers that children and adolescents are constantly growing, therefore it is not possible to assume a single BMI value as a cut-off to define overweight and obesity, as happens for the adult population. 

Several reference standards are available. The International Obesity Task Force has proposed standard centile curves based on pooled international data for body mass index [21]. National growth curves for the Italian population are also available (Italian Society for Pediatric Endocrinology and Diabetes (SIEDP) 2006 growth charts) [22]. However, the most used growth charts are those published by the Centers for Disease Control and Prevention (CDC) for children who are 2 to 20 years of age [23]. The CDC BMI growth charts are the standards that are recommended to diagnose overweight and obesity in children and adolescents ≥ 2 years of age, by the Endocrine Society Clinical Practice Guideline [3]. 

BMI is the accepted clinical standard measure to diagnose overweight and obesity. Nevertheless, it is not a direct measure of body fat, it does not predict the body distribution of fat, and it does not allow the distinction between fat mass and lean mass. Therefore, it could sometimes overestimate adiposity in particularly muscular and athletic children and, conversely, underestimate fat mass in children with reduced muscle mass, such as particularly sedentary patients [17]. 

For the mentioned reasons, during the clinical evaluation, it is important to integrate BMI with other anthropometric parameters. Those include the following:

- Waist circumference (cm) and waist circumference/height ratio (CV/h); this is particularly useful for investigating visceral obesity. A waist/height ratio > 0.5 is indicative of visceral obesity. This parameter is recognized as a better predictor of insulin resistance and metabolic risk in youths [24,25].
- Skinfold thickness; this measure is obtained using a skinfold meter, and evaluates the subcutaneous body adipose tissue by detecting the thickness of the raised skin fold. The triceps skinfold thickness is usually measured [26].

More recently, additional adiposity indices have been proposed, including the body shape index (ABSI), which indicates the abdominal-to-peripheral adiposity ratio, and it highlights the importance of waist circumference in obesity, related to metabolic and cardiovascular complications [27]; the triponderal mass index (TMI), which has been suggested for body composition evaluation and as predictor of MetS [27]; the visceral adiposity index (VAI), identified as a new cardiometabolic risk marker reflecting abdominal fat distribution and dyslipidemia; the conicity index (C-Index), which was recently proposed as a useful tool to screen for MetS and alterations in the lipid profile of adolescents [28].

Bioelectrical impedance, magnetic resonance imaging, and dual-energy X-ray absorptiometry (DEXA), are more technical measurements of body composition assessment. However, such tools are expensive and used much less in routine clinical practice. 

### 3.4. Obesity-Related Complications

The obesity-related complications can occur both in the short term and in the long term. With the global increasing prevalence and severity of pediatric obesity, these overt comorbidities and several initial alterations are beginning to be found in children as well [29].

Obesity is associated to a chronic low-grade inflammatory state, whose effects affect almost all organ systems, with an increased risk of endocrine, cardiovascular, gastrointestinal, reproductive, pulmonary, musculoskeletal, and psychological complications [17,30]. They include hypertension, dyslipidemia, hyperinsulinemia, type 2 diabetes mellitus, cardiovascular disease, nonalcoholic fatty liver disease (NAFLD), nonalcoholic steatohepatitis (NASH), cholelithiasis asthma, sleep apnea, osteoarthritis, hyperandrogenemia, polycystic ovarian syndrome (PCOS), foot and lower limb pain, and musculoskeletal comorbidities [2,31,32].

Obesity is also often related to psychosocial issues, depression symptoms, reduced quality of life, and social isolation [4].

Due to the direct consequences that can occur at pediatric age, it should be added that a high percentage of obese children will carry their excess adiposity into adolescence and adulthood, with a significant increase in the long-term risk of adverse outcomes, especially cardiovascular and metabolic (atherosclerosis, coronary heart disease, metabolic syndrome) [33,34].

The details on the metabolic derangement in a pediatric patient with obesity are detailed in the next sessions. 

**Figure 1:**
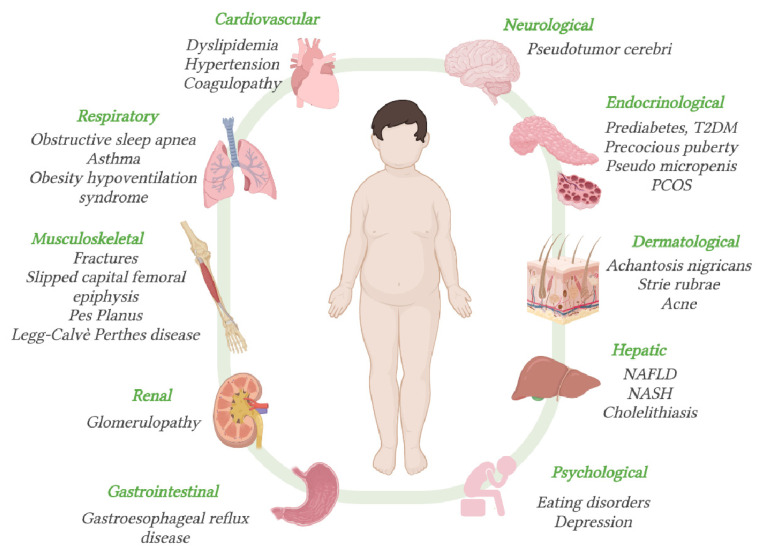
Obesity-related complications. T2DM = type 2 diabetes mellitus; PCOS = polycystic ovarian syndrome; NAFLD = nonalcoholic fatty liver disease; NASH = nonalcoholic steatohepatitis.

The obesity-related complications are summarized in Figure 1.

## 4. Obesity and Metabolic Disorders

### 4.1. Pediatric Obesity and Glucose Disorders

Glucose imbalances are one of the main endocrinologic comorbidities of obesity. Among the glucose dysmetabolisms affecting overweight or obese patients during childhood, it is possible to characterize the following three main entities: insulin resistance (IR), the so-called “prediabetes” state, and T2DM [35,36].

#### 4.1.1. Insulin Resistance

Insulin resistance, in children as in adults, is defined as a state in which insulin produces a subnormal biological response [37]. Thus, the ability of insulin to promote glucose use, by muscle and adipose tissue, is lower than expected, as is its ability to suppress glucose production by the liver [38,39]. 

IR is considered the most common metabolic alteration related to obesity [40]. Recently, a population-based study on IR epidemiology in children has been performed, and the overall prevalence rates of IR ranged between 3.1 and 44%. This difference is mainly due to the different methods that were used to diagnose IR in the studies analyzed, as unanimous diagnostic criteria for this condition are lacking [41]. Among obese children, the prevalence of IR is estimated to be around one third, but it is highly variable, also depending on the different degrees of obesity, racial and ethnic variation, and age of the sample studied [42,43].

Fat tissue itself has a key role in IR development, through the action of different metabolites, adipocytokines, and hormones [37,39]. Among these, adiponectin, one of the main cytokines produced by fat cells, with a fundamental insulin-sensitizing effect, is decreased in obese children [44]. Moreover, it was found that the adipose tissue of obese children is characterized by macrophage infiltration, further highlighting the link between fat tissue inflammation and IR [45,46].

Different inflammatory cytokines and hormonal factors, altered in obese individuals, have been shown to interfere with the insulin signal transduction pathway, leading to IR. Specifically, the cytokines involved seem to be the nuclear factor kb (NF-kB) [47], tumor necrosis factor-α, and interleukin-6 [48]. Some studies also correlated IR to leptin levels [49] in obese children, retinol-binding protein 4 (RBP4) in obese adults [50], and resistin, another adipose tissue molecule, in animal models [48]. Finally, in the development of IR in obese patients, the role of growth factors seems relevant. Indeed, insulin-like growth factors (IGF1 and IGF2) and IGF-binding proteins have been recently correlated to the insulin resistance state in obese adolescents [51].

Further studies are needed to completely understand the precise molecular interactions between these factors that result, initially, in decreased insulin sensitivity, then in overt diabetes. Indeed, IR has been demonstrated to be an important risk factor for the development of DMT2 [39,52], together with β-cell dysfunction [45,53].

#### 4.1.2. Prediabetes

The term ‘prediabetes’ indicates individuals whose glucose levels do not meet the criteria for diabetes, but are too high to be considered normal. The prevalence of prediabetes is, instead, more difficult to determine; it was shown to be around 18% of adolescents, based on data from the National Health and Nutrition Examination Survey [54]. The association of this condition with obesity has been well documented and its incidence was also linearly correlated to obesity severity [55]. Furthermore, overweight children and adolescents have impaired insulin sensitivity, coupled with a declining beta cell function, below the thresholds that are used to define prediabetes states [56,57].

In the spectrum of this condition, the following two different entities are recognized: impaired glucose tolerance (IGT) and impaired fasting glucose (IFG). These are considered to be intermediate stages from the normal carbohydrate metabolism and the overt condition of diabetes mellitus [58].

It is important to highlight that individuals who, according to these criteria, are classified as having an IGT or an IFG, may have glycemic levels in the normal range in daily life, but the importance of these conditions relies in the fact that they are associated with an increased risk of progression to diabetes during adulthood [59].

In this context, it is necessary to underline the fact that prediabetes is often transient during adolescence, as it has been shown that around 60% of prediabetic adolescents return to normal glucose levels in 2 years after puberty. This phenomenon seems to be related to transient insulin resistance, which is a normal component of pubertal development, related to the physiological elevation of the IGF-I/GH axis; plasma IGF-I levels are primarily regulated by GH, which is a counterregulatory hormone that is known to be a potent insulin antagonist [60]. An important risk factor for the persistence of the prediabetic state, and the following development of diabetes, is persistent weight gain [61], and important strategies to prevent this progression are decreased caloric intake and increased physical activities [62].

#### 4.1.3. Type 2 Diabetes Mellitus

Type 2 diabetes (T2D) develops in people with insulin resistance, when the pancreatic β-cell becomes unable to produce sufficient insulin to compensate for the decreased insulin sensitivity. The greater the insulin resistance, the higher the probability to develop overt type 2 diabetes [63]. In obese individuals, blood insulin levels have been shown to be increased in the first phases, and tend to decrease, at sub-normal levels, later in the course of the disease, when β-cell dysfunction progresses [63]. Consequently, at the beginning, the disease progresses silently, evading possible therapeutical interventions, until the deterioration of pancreatic β-cell becomes irreversible and therapy, with medications and/or insulin, is necessary [64].

Many studies have shown that T2D, in the last decades, has tremendously risen in children and adolescents throughout the world [65,66].

The reported prevalence varies according to the different studies, having a range of 1–51/1000, depending upon ethnicity, with higher incidence in Native American, Black, Hispanic, and Asian or Pacific Islander children, with respect to White ones. This trend coincides with the rise in severe obesity in these groups [43,65].

Interestingly, before the mid-1990′s, among children affected by diabetes, only about 1–2% were affected by T2D. Instead, in the last decades, the incidence of T2D has increased to 25–45% of all youths diagnosed with diabetes [67], mirroring the increased incidence of obese children [65].

Overt T2D in children and adolescents is diagnosed according to the ADA criteria [58], with the presence of the typical symptoms of diabetes (polyuria, polydipsia, nocturia, unexplained weight loss) and increased plasma glucose levels, specifically as follows:

- FPG ≥ 7 mmol/L (≥126 mg/dL);
- Post OGTT 2 h plasma glucose ≥11.1 mmol/L (≥200 mg/dL);
- A random plasma glucose ≥200 mg/dL (11.1 mmol/L);
- HbA1c ≥ 6.5% (48 mmol/mol).

The screening for T2DM is only recommended in children and adolescents with specific risk factor for diabetes, of which obesity is the most correlated [58,64].

The long-term prognosis of youth with T2D is not precisely known, but it is estimated that, depending on their glycemic control, these youths may have a decreased life expectancy, by up to 15 years [68]. It seems that glycemic imbalances in young individuals start within two years after diagnosis [35,68] and progress rapidly, increasing the risk of the development of serious health complications, as microvascular and macrovascular diseases, such retinopathy, neuropathy, nephropathy, and cardiovascular disorders [69,70], and the other metabolic comorbidities, including hypertension, dyslipidemia, and fatty liver [70].

### 4.2. Pediatric Obesity and Dyslipidemia

Disorders in the lipid profile are often present in pediatric patients with obesity. According to the population studied, 28% to 46% of overweight and obese children have dyslipidemia [71,72,73,74]. The severity of obesity may influence the prevalence of an abnormal lipidic profile in a given cohort. The risk for an obese child to have dyslipidemia was calculated to be 2.8× higher than for a child with a normal BMI [71].

The most common dyslipidemic pattern that is associated with childhood obesity is secondary combined dyslipidemia, known as combined dyslipidemia of obesity (CDO). The disorder presents a combination of moderate-to-severe elevation in triglycerides and non-high-density lipoprotein cholesterol (non-HDL-C), mild elevation in total cholesterol (TC) and LDL-cholesterol, and low HDL-C level [71,75,76].

When evaluating a child for combined dyslipidemia, it is useful to add the following two significant measures to the routine lipid panel: non-HDL-C and serum triglyceride-to-HDL cholesterol ratio (TG/HDL-C ratio) [76].

Non-HDL-C proved to be a good predictor of subclinical atherosclerosis in asymptomatic younger adults [77].

The TG/HDL-C ratio has been shown to be solidly related to the atherogenic lipid profile, coronary disease development risk, and extent of coronary disease in adults [78]. TG/HDL-C ratio > 2.2 (corresponding to the 75th percentile) can be considered a useful and simple marker of atherogenic dyslipidemia and an altered cardiometabolic risk profile in Italian children with obesity [6,78].

As reported by the Princeton LRC Follow-up Study, 14.6% of subjects with hypertriglyceridemia in both childhood and adulthood had CVD, compared to 2.9% of subjects with high triglycerides (TG) only during adulthood, and 1.9% of subjects with high TG when they were children and then normalized in adulthood. Instead, the incidence of CVD was 1% in the group without a story of past or present hypertriglyceridemia [79].

The dyslipidemic pattern has been shown to be linked to the initiation and progression of atherosclerotic lesions in children and youths [80]. The Bogalusa Heart Study clearly pointed out how fatty streaks, an early sign of atherosclerosis, are commonly found in the aorta and coronary arteries autopsies of children aged 2–15 years, who did not die from cardiovascular causes [81].

It is known that excess circulating low-density lipoproteins (LDL) accumulate in the vascular internal wall, and are oxidized (oxLDL) and phagocytized by macrophages, which transform themselves into foam cells and give origin to the fatty streaks. However, other factors are involved in atherosclerosis too, and inflammation has a key role [82].

A Bogalusa Heart Study analysis also showed that the extent of fatty streaks covering the intimal surface of the vessels increased in young people with multiple risk factors (high BMI, systolic blood pressure, LDL-C and triglycerides), supporting the notion that risk factors exert a synergistic effect on cardiovascular events [81].

Obesity is a state of chronic low-grade inflammation and is typically characterized by dysfunctional changes in the adipose tissue, in terms of both the adipocytes’ size and profile secretion [83]. Under the obese status, the hypertrophied adipocytes show altered adipocyte-derived cytokine production and metabolic derangements.

For instance, obese patients have lower circulating levels of the anti-inflammatory adiponectin and omentin [84,85,86,87], and increased circulating levels of both various proinflammatory cytokines and hormones (such as leptin, resistin, IL-6, IL-10, tumor necrosis factor α TNF-α) [86,88,89]. The unbalanced adipokines secretion may affect the cardiovascular system, thus contributing to explaining the obese-related cardiometabolic disorders [83]. Adipose tissue from obese individuals is in M1-like macrophages, which secrete large amounts of TNF-α and other pro-inflammatory molecules. Conversely, the expression of adiponectin is reduced. Adiponectin has vascular protective functions, mainly mediated through the activation of endothelial nitric oxide synthase (eNOS), and insulin-sensitizing properties [90].

Obese patients often have signs of insulin resistance (IR). Thus, insulin antilipolytic action is impaired and IR promotes free fatty acid (FFAs) release from the adipose tissue into the portal circulation, excess FFAs flux to the liver and undergo hepatic accumulation [91]. It has been proposed that high FFAs levels and inflammatory cytokines diminish nitric oxide (NO) production by eNOS, leading to endothelial dysfunction. Other alterations, such as increased expression of plasminogen activator inhibitor-1 (PAI-1, a prothrombotic molecule) and increased vascular smooth muscle cell proliferation, follow the decreased NO levels, and contribute to creating the proinflammatory environment that promotes atherosclerosis [92,93].

Moreover, the increased FFA flux to the liver increments hepatic triglycerides (TG) synthesis and the secretion of VLDL. Insulin is a stimulator of lipoprotein lipase (LPL), which is the main enzyme involved in the hydrolysis of TGs. Consequently, LPL action is impaired in the insulin-resistant status. Excessive TG deposition in the liver and peripheral tissues promotes, in turn, insulin resistance, in a vicious circle.

Lastly, elevated TG concentrations are associated with increased levels of proatherogenic small, dense LDL particles and reduced levels of HDL-C [91,94].

The management of children with a dyslipidemic pattern should start with early detection.

As for dyslipidemia, the Consensus Position Statement of the Italian Society for Pediatric Endocrinology and Diabetology recommends assessing cholesterol, HDL-cholesterol, and triglycerides, in all children and adolescents with obesity since the age of six. The measurements should be repeated every three years, or sooner if there are abnormalities or the child develops comorbidities or rapid weight gain [6].

The American Academy of Pediatrics and the American Heart Association endorse the National Heart, Lung, and Blood Institute (NHLBI) expert panel guidelines, which recommend universal routine lipid screening for all children and adolescents of 9–11 and 17–21 years. Targeted screening is strongly suggested for children of 2–8 years and 12–16 years, with risk factors for dyslipidemia (including diabetes, hypertension, smoking, BMI ≥ 95th percentile for age and sex if 2–8 years, BMI ≥ 85th percentile for age and sex if 12–16 years, positive family history for cardiovascular diseases) [75].

The diagnosis of dyslipidemia is based on the criteria advanced by the NHLBI expert panel, adopted by the Italian Consensus Position Statement too. The references values that are considered acceptable, borderline-high, and high, are listed in Table 1 below [75].

The lifestyle interventions focused on diet and physical activity, proposed as the first-line dyslipidemia treatment in childhood [75].

## 5. Therapeutic Strategies for Pediatric Obesity: The Lack of Effectiveness of Current Treatments in Weight Loss and Dysmetabolism

The prompt treatment of overweight/obese children and adolescents is extremely important, as this condition affects many systems and has negative consequences both at the physical and at the psychological level. Thus, the therapy should have a multidisciplinary approach [1,95]. The therapeutic strategies can be divided into the following three main groups: non-pharmacological treatment, pharmacological ones, and, lastly, bariatric surgery [95].

### 5.1. Non-Pharmacological Treatment

The fundamental pillar of the non-pharmacological treatment is education. Indeed, both the patients and their families should be instructed to correct their lifestyle changes, mainly focused on a healthy diet and an increase in physical activities [5].

Before the eventual start of a personalized diet, families are educated to a heathy diet, with reduced sugary and industrialized foods or beverages, as well as products that are high in fats, of animal origin. Moreover, an increased dietary intake of fruits and vegetables is promoted, as these strategies have shown a relevant effect in weight loss [5]. A balanced and high-fiber diet, during childhood and adolescence, has also been correlated to improvements in the glycemic control later in life, as it increased peripheral insulin sensitivity and lowered fasting glucose levels [96,97]. Importantly, these nutritional habits must be linked to daily moderate physical activity, tailored on the patient’s age, tolerance, and preference, to increase the compliance as much as possible [98,99].

The recommended amount of physical activity is a minimum of 20 min per day [3], of moderate-intense activity, correlated to an increase in insulin sensitivity, independently from of the body fat percentage [100]. Regular vigorous physical activity was shown to influence insulin action on skeletal muscle glucose and fat metabolism [101], but the mechanisms behind the decreased IR have not been clearly elucidated yet [102].

The amount of weight loss is individualized according to the patient’s age, obesity severity, and eventual comorbidities [103]. Moreover, family-based behavioral approaches are recommended, as shown, to give better results [5,104].

There is still lack of consensus concerning the best structured diet for weight reduction in overweight children and adolescents. Low-carbohydrate and low-glycemic index (GI) regimens have been shown to be comparable, in terms of short-term weight loss, to standard portion-controlled diet [105,106].

It was recently demonstrated that in obese/overweight adolescents, the lack of effect of a high-protein/low-glycemic index diet on BMI reduction and IR was mainly due to insufficient dietary compliance after 2 years [107].

Because of the scarce results that have been obtained with lifestyle interventions, which rarely result in long-term weight loss and resolve obesity-associated comorbidities [108], the use of a pharmacological approach may be needed [1,5].

### 5.2. Pharmacological Treatment

The pharmacological treatment of childhood obesity is still a debated theme [5,109]. Importantly, pharmacological management is suggested only after the unsuccess of a formal program of lifestyles changes [1]. Indeed, the main drugs that are used in this field are orlistat, approved for obese adolescents ≥ 12 years old by the Food and Drug Administration [110], phentermine, a sympathomimetic amine, used for the short-term management of obesity in individuals > 16 years of age [111], liraglutide, an agonist of the glucagon-like peptide-1 receptor, and metformin, approved for the treatment of children ≥10 years old, who are affected by DMT2, and used off-label for weight managements in pediatrics [112,113].

In more detail, orlistat is a potent lipase inhibitor, which acts at the gastrointestinal level, blocking up to 30% of the absorption of fats from the diet. Unfortunately, the efficacy of this drug, in terms of loss of weight, is only modest, and it has several gastrointestinal adverse effects, such as diarrhea, flatulence, and fatty stools [110]. These aspects, coupled with the risk of fat-soluble vitamin deficiencies and the consequent need of multivitamin supplementations, limit its use [109].

Metformin, instead, is the drug of choice for diabetic children and obese adolescents. This drug decreases the glucose production from the liver, increasing peripheral insulin sensitivity, and thus decreasing IR [62,99]. Metformin is extremely useful and effective in the management of glycemic metabolism [58]. Indeed, it was shown to improve glucose homeostasis in obese insulin-resistant children, and delay the appearance of impaired glycemic metabolism in children who were at high risk for DMT2 [114], but when administered to promote weight loss, it only resulted in modest reductions in BMI [115].

The pharmacological treatment of choice for lipid dysmetabolisms in obese children are statins, HMG-CoA reductase inhibitors. These drugs are recommended, according to the American Association of Pediatrics (AAP), in addition to lifestyle changes, only in patients ≥8 years old with LDL cholesterol ≥ 190 mg/dL, or ≥ 160 mg/dL with risk factors. In the case of the presence of DM, therapy can be started with LDL cholesterol ≥ 130 mg/dL. According to the National Heart Lung and Blood Institute (NHLBI), instead, therapy can be started in children ≥ 10 years old with LDL cholesterol consistently ≥ 190 mg/dL. In children ≤8 years old, instead, the pharmacological therapy is started only in the case of an LDL cholesterol higher than 500 mg/dL, according to the American Association of Pediatrics [75], and in children ≤ 10 years old, who are affected by severe primary hyperlipidemia or a high-risk condition associated with severe medical morbidity, according to NHLBI [116].

### 5.3. Surgical Treatment

The last option for obesity management is bariatric surgery. Specifically, bariatric surgical procedures are Roux-en-Y gastric bypass, biliopancreatic diversion with duodenal switch, adjustable gastric banding, and laparoscopic sleeve gastrectomy [117]. The vertical sleeve gastrectomy is the procedure of choice for severely obese adolescents, according to the Pediatric Metabolic and Bariatric Surgery guidelines [118,119].

This approach, in the adult population, resulted in a significant and sustained BMI decrease and was also correlated to improvements in obesity-related comorbidities (such as DMT2 and lipidic dysmetabolism), as well as a reduction in mortality [120,121].

Bariatric surgery, in the pediatric patients, is reserved only for cases in which lifestyle changes and pharmacological treatment approaches are not effective in weight loss and the control of comorbidities [122]. Importantly, for patient selection, strict criteria must be respected, according to the American Society for Metabolic and Bariatric Surgery [118].

The surgical approach in adolescents, independently from the surgical techniques, was shown to be beneficial, in terms of metabolic unbalances, as dyslipidemia and DMT2, and BMI reduction [122].

Bariatric surgery leads to more significant weight loss in severely obese adolescents, with respect to lifestyle interventions [119]. Moreover, it was correlated to an improvement in the quality of life [123,124].

Although effective, both in terms of weight loss and cardiovascular risk factors reduction [122], surgical procedures are invasive and have important complications both in the short term (such as wound infections, anastomosis leakage, and bowel obstruction) [125] and in the long term (deficiencies in thiamine, vitamin b12, iron, and vitamin D) [126]. Thus, lifelong vitamin and mineral supplementation is recommended, but, unfortunately, also in this case, the adherence to recommendations is scarce [5].

The long-term success of the current treatments is still limited, as is that of the current prevention strategies [127,128], thus the negative impact of obesity-related comorbidities remains an extremely important health issue [129].

A new approach is indeed needed to face childhood obesity, which is defined as one of the most important public health problems in the world [5].

## 6. Ketogenic Diet: Indications in Infants and Children

The ketogenic diet (KD) is an established non-pharmacological treatment that is used for infants and children with drug-resistant epilepsy (DRE) [130,131,132]. It consists of a high-fat, low-carbohydrate and adequate-protein diet, designed to mimic the effects of fasting on the organism [132]. Fatty acids are used as the main energy source, through the production of ketones, resulting in improved inhibitory neurotransmission and decreased seizure frequency [133].

### 6.1. Ketogenic Dietary Therapies in Infants and Children

A 2016 Cochrane review reported that KD is a viable option in patients with intractable epilepsy or who are unsuitable for surgery [134].

KD was traditionally not recommended in children under 2 years of age. Although, KD has been reported to be a safe, effective, and a practical management modality in breastfeeding infants and children under 2 years of age, with drug-refractory epilepsy [130,135,136].

In recent years, there has been a great increase in the interest and research suggesting similar results in the use of dietary therapies for adults [137].

Ketogenic dietary therapies are the treatment of first choice for some metabolic disorders and types of epilepsy (Table 2). Nevertheless, KD is contraindicated in several specific inborn errors of metabolism that could lead to a severe metabolic crisis in children. Relative and absolute contra-indications should, therefore, be ruled out before starting the diet (Table 2) [130,131,138].

The implementation of KD is challenging, but the primary outcome of crisis reduction is generally achieved [139,140].

Acceptance of this modality, finding the essential tools, and adhering to a dietary regime, can affect the quality of life (QoL), and this is the major disadvantage of this new therapeutic frontier. Close support and motivation for the family is required. A systematic review of the literature assessed the effect of KD on the QoL of their immediate family members [141].

Other negative features and reasons for discontinuation are major adverse effects. Gastrointestinal adverse effects, particularly vomiting, diarrhea, and constipation, are the most common, and occur in 30% of patients during the initiation phase. Overall, they responded to dietary adjustments and medication, and therefore this did not lead to discontinuation of the dietary treatment [133]. Up to 7% of children on KD therapy may develop kidney stones [142]. QT interval prolongation and cardiomyopathy have been found during prolonged KD, with unclear causal mechanisms [143]. Osteoporosis and vitamin D deficiency may be observed in children on KD, especially when these children are on multiple anti-seizure medications [144]. Biochemical alterations that may be observed with KD include hypercholesterolemia, hypertriglyceridemia, and depressed levels of low-density lipoprotein (LDL) [145].

Clinical evaluation and screening laboratory studies should be obtained prior to starting KD, in order to identify the seizure type, rule out metabolic disorders, and evaluate for complicating comorbidities [138,146]. Dietary therapy should be provided for at least 3 months before considering that the therapy is ineffective. All the children should receive a daily multivitamin, calcium, and vitamin D intake. Oral citrates appear to prevent kidney stones; however, there is still no unanimous consensus on its use. There is no recommendation for the empiric use of antacids, laxatives, probiotics, exogenous ketones, additional selenium, or carnitine, with the KD currently [138].

### 6.2. Ketogenic Diet: Nutritional Composition

The ketogenic diet is a nutritional protocol, with high fat (70–90% energy), carbohydrate restriction (4–19%), and an adequate amount of protein to support growth [130]. The ketogenic diet is calculated in grams of fat to grams of proteins plus carbohydrates. This “ketogenic ratio” varied among different types of KD and may range from 1:1 to 4:1 [131]. Therefore, the permitted quantity of carbohydrate follows from the calculation of energy and protein requirements, and the establishment of the necessary quantity of fat [130]. Reaching an adequate protein intake is recommended in infants, which the daily RDA have to be respected in order to sustain growth [130,131]. Nowadays, five types of KD, with different compositions, are currently prescribed in clinical practice [147] (Figure S1 in Supplementary Materials).

The classical version of KD is based on a 4:1; this means that for every 4 g of fat, there is 1 g of combined protein and carbohydrates. In this classic protocol, 90% of the energy comes from fats, usually long-chain triglycerides, and only 10% from protein and carbohydrates [138,148]. The traditional method of initiating KD involves a period of fasting (12–48 h), after which food may be progressively administrated. Fasting can result in hypoglycemia, acidosis, dehydration, and lethargy, therefore the classic KD protocol was usually instituted in hospitals [131]. Nowadays, fasting is no longer required and recommended as well as initial fluid restriction; indeed, a gradual initiation protocol offers the same seizure control compared to a traditional KD protocol [131,147].

Moreover, a 3:1 ratio can be used alternatively, to increase the protein and carbohydrate intake [130]. This ketogenic ratio is more appropriate in infants, not only for diet initiation, but also in order to meet the protein requirements [130]. Different studies showed that a lower ketogenic ratio is as effective as a 4:1 ratio, and less side effects can be observed [133,149,150].

Particularly in young infants (<12 months), diet initiation should be undertaken without fasting and with a stepwise start, starting with a 1:1 ratio and progressively reaching the level of ketosis required. In addition, a KD formula with ratio 3:1 can be used purely or combined with breast milk [130,138].

Despite being the conventionally prescribed diet, the classic KD has an highly restrictive nature, which may require hospitalization at the outset [147]. Moreover, classic KDs are associated with poor compliance, thus lowering the ratio may improve the compliance, reducing the difficulties to follow this approach [138].

Another approach is the KD with medium-chain triglycerides (MCT), which comprises 60% of the energy from MCTs. However, the use of MCT in infants is limited. Older infants can use 50% MCT mixed with a low-fat milk product, or, alternatively, a low-amount (20% MCT) emulsion drink. Usually the tolerability of the total MCT in infants is 10–25% of the daily energy intake. Indeed, a high level of these fats can cause common adverse effects, such as abdominal discomfort and bloating, which lead to the discontinuation of therapy in most children [130,147,151].

This protocol has an increased ketogenic potential, as MCT are absorbed more rapidly than long-chain triglycerides [152]. As a result, an MCT-based diet yields more ketones per calorie of energy, resulting in a less total fat requirement compared to classic KD, thus allowing consumptions of more carbohydrates and proteins [131,152,153]. It is comparable to the classical KD in efficacy and tolerability, with it also being less restrictive and more palatable [154].

The modified Atkins diet (MAD) is a less restrictive KD, in which fat provides approximately 65% of the calories, with approximately a 1:1–1.5:1 ketogenic ratio [155]. The advantages of no limitation on protein, fluids, or calories, make this protocol easier to follow [138], in which the initiation does not require prior hospitalization and restriction of other micronutrients [131,153]. MAD is effective in children with refractory epilepsy, over 2 years of age and with Lennox-Gastaut syndrome [156,157]. However, classical KD was significantly more successful in children under two years of age [157]. Currently, it is a promising choice for resource-limited settings, but in developed settings it is predominantly used in adolescents and adults [147].

Finally, low-glycemic index (LGI) diets are based on a nutritional protocol that is focused on lowering the glycemic index of foods in the diet. LGI diets aim to flatten the postprandial blood glucose and insulin curves, and their positive effect has been proved in obesity, diabetes, and other non-communicable diseases [158,159,160]. This model is also suitable to the ketogenic diet, which is a less restrictive form of ketogenic therapy (LGI ketogenic diet), composed of 60% fats, 10% carbohydrates, and 30% proteins [161]. The amount of carbohydrates intake is approximately 40–60 g/day, but of low-glycemic index indices (<50). An LGI-based diet produces minimal ketosis compared to classic KD, with equivalent anti-seizure efficacy and a better safety profile. This variant of KD has been found to be particularly effective in controlling seizures in patients with Angelman syndrome [147,162].

**Figure 2:**
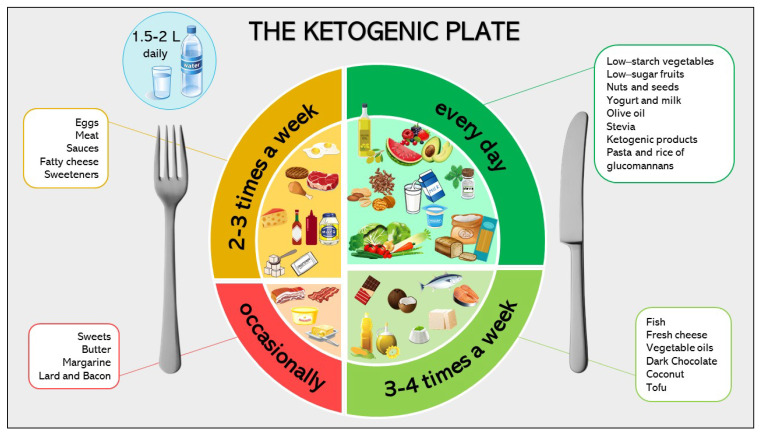
The ketogenic plate.

The dietary choices for children and adolescents following a ketogenic diet are summarized in the “ketogenic plate” (Figure 2).

### 6.3. Ketogenic Diet: Mechanism of Action

Nutritionally induced ketosis is the condition where fatty acid oxidation is diverted to ketone production in the liver, because of low tissue glycogen levels [163]. When the glucose reserves run out, the central nervous system (CNS) is not able to use fatty acids as an energy source, thus, ketone bodies, produced from acetyl-CoA, are produced as alternative energy sources. Since, in postnatal life, these molecules are essential during brain formation, acting as lipid precursors and sparing glucose [148]. Although rapid, the ability of the brain to switch from one energy source to another requires significant metabolic adaptation, including the changing of ketone bodies transporters in response to ketone level variations [148,164]. Moreover, KD is significantly effective in children, due to the greater permeability of the blood barrier.

Many cell types in the CNS are able to use ketone bodies as a glucose substitute, and are spared the damaging effects of glucose deprivation. In particular, beta-hydroxybutyrate (βHB) is the primary energy source for neurons, if glucose is compromised [148,165,166]. However, according to studies that examined the effects of ketone body supplementation to glucose, under cell culture conditions, ketone bodies are preferentially used in lipid synthesis, while glucose remains the primary energy source in the CNS, when feasible [148,167].

**Figure 3:**
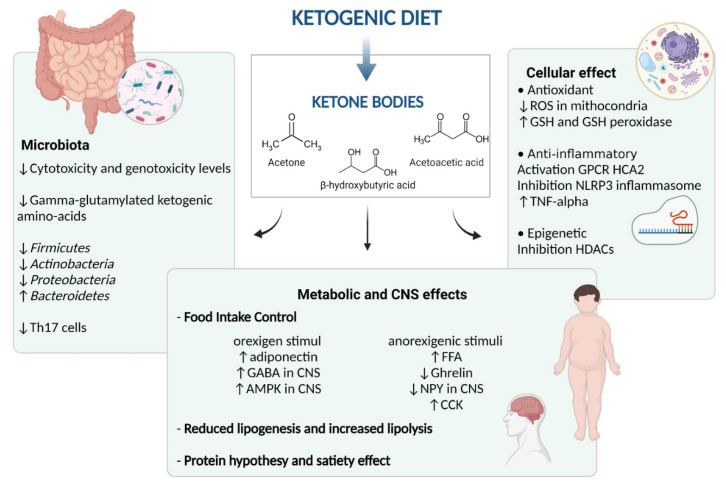
Mechanisms of ketogenic diet action. ROS = reactive oxygen species; GABA = gamma-aminobutyric acid; GSH = glutathione; G-protein-coupled receptor (GPCR); HCA2 = hydroxycarboxylic acid receptor 2; HDAC = histone deacetylase; Th = T helper; TNF-α = tumor necrosis factor alpha; NPY = neuropeptide Y; CCK = cholecystokinin; CNS = central nervous system; AMPK = AMP-activated protein kinase.

There is supportive evidence that the ketogenic diet exerts an anti-seizure effect, through different mechanisms, having the CNS as the main focus of action. Although the mechanisms underlying the effects of KDs on weight loss are still a subject of debate [168]. There are multiple potential mechanisms through which the ketogenic diet may affect the obesity phenotype and status (Figure 3).

Gibson et al., in their metanalysis, reported that the clinical benefit of a ketogenic diet relies on/stays in preventing an increase in appetite, despite weight loss, although individuals may indeed feel slightly less hungry (or more full or satisfied). Ketosis appears to provide a plausible explanation for this suppression of appetite [169]. A possible explanation underlying this mechanism of action is the suppressant action of ketone bodies on appetite or modifications in hormone control.

In more detail, ketosis might have a direct or indirect effect on the secretion of appetite-related hormones, as they seem to exert an action on both orexigen and anorexigen signals, mainly (βHB) [163,170]. In the orexigen mechanisms, KD increases the circulating levels of adiponectin, while acting in the CNS, regulating food behavior, via enhancement of the brain gamma-aminobutyric acid (GABA) and AMP-activated protein kinase (AMPK) phosphorylation. The anorexigenic mechanism implies a rise in the circulating free fatty acids after meals, and a subsequent reduction in NPY, which is a neuropeptide that is essential in the food intake control, acting on the arcuate nucleus (ARC) of the hypothalamus. Moreover, KD decreases the circulating ghrelin levels (the appetite hormone) and maintains the CCK post-prandial anorexigenic response after weight loss. Thus, the net balance of the contrasting stimuli results in a general reduction in perceived hunger and, consequently, lower food intake [163,170].

Following this, KD modifies the metabolic pathways of the subject, by reducing lipogenesis and increasing lipolysis, thus influencing the adipose tissue and dyslipidemia status. Moreover, another hypothesis suggests the positive role of protein intakes, of which the utilization by the body requires an “expensive” process that increases energy expenditure compared to other diet protocols. In fact, glucose production during KD is obtained from the gluconeogenesis pathway, an energy-demanding process, which relies on dietary or tissue-origin proteins [168]. Together with this, the protein hypothesis states possible mechanisms for higher weight, due to the higher satiety effect of this nutrient [8,168].

The ketogenic diet has beneficial effects on the obese phenotype, which are broader than simply fat and weight loss. Obesity is a recognized systemic low-grade inflammatory state, in which adipose tissue iper-proliferation alters the signaling between the adipocytes, immune cells, and epithelial cells. Thus, resulting in a secretion of active molecules, such as cytokines, TNF-alpha, and IL-6, which increase insulin resistance and stimulate inflammatory processes [171]. Given that KD is a potential tool in counteracting inflammation, for example, by reducing TNF-alpha after treatment [172].

Furthermore, ketone bodies are useful antioxidative and anti-inflammatory molecules at the cellular level. In fact, the ketogenic diet has been associated with antioxidant effects, producing lower amounts of ROS (reactive oxygen species) in the mitochondria, and increasing glutathione (GSH) and glutathione peroxidase activity in animals [165]. Recently, βHB has been found to modulate inflammation through the following two mechanisms: via activation of Gi-protein-coupled receptor HCA2, contributing to the neuroprotective effect and via the inhibition of NLRP3 inflammasome, which mediates IL-1β and IL18 production in human monocytes [173,174].

Lastly, ketone bodies could exert epigenetic cellular effects, involving the inhibition of histone deacetylases (HDACs) enzymes, and consequently affecting transcription differently and upregulating some genes encoding for bioenergetic enzymes [165,174,175].

### 6.4. Ketogenic Diet and Gut Microbiota

The gut microbiota and their metabolites have gained attention as possible players that directly modulate host health [147,176]. SCFA, namely, butyrate, acetate, and propionate, are gut metabolic end-products that exert multiple beneficial effects on human metabolism, particularly in obese individuals [176,177]. Recently, studies [10] have shown changes in the gut microbiota, after adopting the ketogenic diet, suggesting a possible role of ketone bodies in altering the intestinal microbiome. Interestingly, recent preclinical data underline the possible pathogenetic role of the gut microbiome on the benefits of the KD [178].

In mouse models receiving KD, an increase in *Akkermansia muciniphila* and *Parabacteroides merdae*, in the intestinal population, has been noted. These bacteria reduce gamma-glutamylated ketogenic amino acids, both in the gut and blood. This fact, in turn, had the effect of rising the ratio of GABA-to-glutamate in the brain of mice. Therefore, the reduction in GG amino acids, displayed by the gut microbiota, might be linked with neurotransmission at the CNS level [179].

Moreover, recent studies suggested that ketone bodies inhibit the reduction in the levels of proinflammatory Th17 cells in the intestine, therefore acting as possible modulators of inflammatory conditions [132,180].

Clinical evidence on the children population is still limited to epileptic patients. For example, a group of 14 epileptic children, evaluated before starting the KD diet, experienced an imbalance in the gut microbiota compared with healthy controls, harboring a higher amount of pathogenic bacteria and decreasing beneficial bacteria. After a KD treatment, a healthier microbiota was observed, by decreasing the *Protobacteria* (from 24.34% to 10.77%) and enhancing the *Bacteroidetes* phylum (from 26.75% to 38.71%) [181]. According to these, Zhang et al. also found a selective increase in *Bacteroidetes*, and a decrease in *Fimicutes* and *Actinobacteria*, after KD treatment in epileptic children [182].

Interestingly, applying KD in a group of obese adults, Basciani et al. observed a changed microbiota pattern, which resembled the ones observed in children who are affected by refractory epilepsy, treated with KD, i.e., *Fimicutes* significantly diminished and *Bacteroidetes* increased. Moreover, they found divergent responses on the gut microbiota, according to a protein source during KD, experiencing a healthier microbiota composition with whey or vegetable protein sources in the diet [183].

Due to the low fermentable carbohydrate intake during KD, gut metabolites significantly reduce during the ketogenic diet treatment. Therefore, Ferrari et al. studied the impact of this high-fat diet protocol on gut health, having expected an increase not only in the bile acid secretion, but also in the secondary bile acid detrimental effects. Surprisingly, the KD diet, even though it is a high-fat protocol, showed a decrease in cytotoxicity and genotoxicity levels. The absence of adverse effects on fecal water toxicity after KD treatment, has therefore been assigned to the better health conditions of patients after the KD diet [184]. The hypothesis of a multistep impact of KD on human health, besides the gut environment, to a systemic level, is still open.

Given that obesity is a well-known condition, in which the Firmicutes/Bacteroidetes ratio is altered, KD is gaining attention as a possible therapeutic strategy, to ameliorate the inflammatory condition by changes in the gut microbiota species and metabolites [176,182,185,186].

## 7. Ketogenic Diet and Metabolic Disorders in Adults and Children: State-of-the-Art

Indications and contraindications for the use of VLCKD diets in adults, are shown in Table 3 [187,188]. VLCD and VLCKD diets are administered orally and often using commercial products that contain nutrients of high biological value, and may be in solid, liquid, or powder form. Recently, EFSA expressed a scientific opinion about the correct amount of macronutrients, establishing a minimum content of protein (75 g/day), carbohydrates (30 g/day), linoleic acid (11 g/day), α-linoleic acid (1.4 g/day), and not less than 600 kcal/day, until 800 kcal/day. In addition, VLCKDs can be followed for 12 consecutive weeks, under medical supervision [189].

In recent years, VLCDs had been explored for the treatment of several diseases in adults, proving a number of potential therapeutic impacts on the gut microbiota, cancer, diabetes, weight loss, and cardiovascular diseases [190].

The condition that sets in, following a few days of adherence to VLCKD, is known as ‘physiological ketosis’, which, unlike ketoacidosis that is caused by metabolic decompensation, maintains a physiological pH and low levels of ketones [191]. The subjective sensation of appetite is greatly reduced during this diet, probably in relation to the suppression of ghrelin secretion, which is the main gastrointestinal estrogen hormone [192]. Finally, no clinically relevant changes in liver function have been reported during VLCKD, and this diet can be considered safe [193]. The most frequently reported side effects are headache, halitosis, constipation, alternating with diarrhea, electrolyte disorders, and muscle cramp [187].

After the most restrictive phase, patients can gradually reintroduce different food groups (starting with those with a lower glycemic index, then moderate, and, finally, high), and in the meantime they follow a nutritional re-education program, to promote a change in their eating habits and to maintain weight loss in the long term [191].

### 7.1. Ketogenic Diet and Obesity

A 2014 systematic review and meta-analysis of 20 RCTs, conducted on adult patients aged 28–48 years, with a BMI between 27.9 and 41.9 kg/m^2^ (from overweight to severe obesity), found significant weight reduction during the VLCD and low-calorie diet (LCD) adherence period. In contrast, in the maintenance phase, and up to 22 months after following the diet, less weight regain was observed in the anti-obesity drugs group or meal replacements group, compared to a protein-rich diet group or exercise group [194].

In addition, a 2017 review and meta-analysis of five rcts, assessing weight loss in diabetic and non-diabetic patients after following VLCD (<800 kcal/day) or low-energy liquid-formula diets (LELD) (>800 kcal/die), found that the weight loss was the same in both the diabetic and non-diabetic patients, ranging from 8 to 21 kg, over a period of 4 to 52 weeks.

Moreover, a recent systematic review and meta-analysis of 15 studies (7 noncontrolled, 2 controlled, and 6 randomized controlled studies) shows significant reduction in body weight and BMI at 1, 2, 4–6, 12, and 24 months, and this and appears to be associated with larger weight loss rates compared to other diets with a different energy content (i.e., LCD and VLCD) of the same duration [187]. They also found a reduction in waist circumference and a loss of lean mass, comparable to that found in subjects who follow other dietetic interventions [187].

A recently published narrative review explores the various positive effects of the ketogenic diet [190]. In particular, on weight loss, they report a study [195] that was conducted on 322 moderately obese patients, over a period of two years, following a low-fat restricted-calorie diet (LFD), a Mediterranean restricted-calorie diet (MD), and low-carbohydrates non-restricted calorie diet (LC). The LC group was instructed to reduce carbohydrates (<20 g/day) for the first two months, and then increase to up to 120 g/day of carbohydrates. The weight loss after 3 months was greater in the low-carb group, but when carbohydrates were reintroduced, the weight was similar to the MD group. A similar study [196] monitored, for two years, weight loss and changes, in visceral fat, using DEXA. The participants were divided into the VLCKD group and LCD group. The weight loss, in kilograms, in the VLCK diet was double that of the LC diet throughout most of the study, and remained significant at the end of the trial. The amount of visceral fat loss in the VLCK diet group was three times greater than the control group, while preserving lean body and skeletal bone mass [196]. This evidence, regarding the maintenance of lean mass, is a fundamental point, because when rapid weight loss occured, the basal metabolic rate (BMR) decreased, leading to weight regain, due to increased hunger and lower energy expenditure.

### 7.2. Ketogenic Diet, Insulin Resistance, Type 2 Diabetes and Polycystic Ovary Syndrome

Correct nutrition should be considered to be an integral part of the metabolic management of diabetes, and the ketogenic diet could at least be offered as a treatment option [197]. The rapid reduction in carbohydrate intake reduces glucotoxicity and insulin resistance, improves pancreatic beta-cell function, and leads to better glucometabolic control [198].

In patients undergoing a VLCKD, hepatic gluconeogenesis maintains stable glycaemia through basal insulin secretion. In fact, the preserved insulin secretion prevents the onset of pathological ketoacidosis. The differences between the normal diet, the ketogenic diet, and diabetic ketoacidosis, are summarized in Table 4 [168].

Over the years, several scientific societies have expressed positions on the role of the ketogenic diet in diabetes. In 2019, a consensus was published that considering reducing the overall carbohydrate intake, with low-carbohydrate (LC) or very-low-carbohydrate (VLCD) meal plans, is a feasible approach for adults with type 2 diabetes, who are not achieving their glycemic targets or where reducing hypoglycemic medication is a priority [199]. The American Diabetes Association’s Position Statements 2020 also suggested the use of a low-carbohydrate meal plan for people with prediabetes, stating that further research is needed to establish its usefulness [200,201]. Numerous systematic reviews and meta-analyses of RCTs on patients with DMT2, show that both VLCD and VLCKDs induce greater weight loss in the short term (<6 months) than standard hypocaloric diets. The most important aspect, however, seems to be the duration of the positive effects on glucometabolic set-up. Steven et al. confirmed that recent-onset TMD2 can be considered to be characterized by a reversible, altered β-cellular response, and thus that the first phase of insulin secretion can be recovered. VLCD was particularly effective in patients with a short duration of diabetic disease and a preserved insulinemic response. The patients who followed VLCD for 2 months showed weight reduction and stable insulinemic levels at 6 months. After returning to an isocaloric regimen, a lower fasting blood glucose was observed without the use of hypoglycemic drugs. [202]. A recent systematic review, using American Diabetes Association remission definition (<6.5% HbA1c threshold), found that patients who adhered to VLCD resulted in 32% increase rates of remission of diabetes at 6 months, compared with diets that are commonly recommended for DMT2 management [203]. In terms of body composition, to support the efficacy of this treatment, lean mass and fat mass were assessed by X-ray densitometry (DXA), basal metabolism by indirect calorimetry and biochemical analyses for metabolic balance, in 25 subjects with TMD2, who followed VLCKD for 8 weeks. The results show reduced abdominal fat mass, maintenance of resting energy expenditure (REE), and restoration of metabolic function [204].

The different studies show that the duration of the treatment and the gradual transition to the different regimes is crucial for positive results. After the planned weeks of VLCKD (600–800 kcal/day, CHO 20–60 g/day), the transition phase begins, during which carbohydrates are gradually reintroduced. Foods with a lower glycemic index (fruit and dairy products) are the first to be reintroduced, followed by foods with a moderate glycemic index (legumes), and finally foods with a high-glycemic index (bread, pasta, and cereals). During the transition phase, the quantity of carbohydrates should not exceed 90 g/day and the daily calorie intake should not exceed 1500 kcal. In the subsequent maintenance period, the quantity of carbohydrates should not exceed 130 g/day and the calorie intake should be between 1500 and 2000 kcal/day. The main purpose should be to maintain weight loss and promote a healthy lifestyle, as close as possible to the fundamentals of the Mediterranean diet [205,206].

Insulin resistance and obesity are common signs of polycystic ovary syndrome (PCOS). Hyperinsulinemia contributes to hyperandrogenism in women with PCOS, which, in turn, is responsible for increased visceral and subcutaneous body fat [207]. Interestingly, low-grade inflammation, with an excess of carbohydrate intake, acts with insulin resistance and hyperandrogenism, to enhance the metabolic phenotype of PCOS [208]. Acute hyperglycemia produces reactive oxygen species (ROS), and increases oxidative stress and inflammation [209]. Blood glucose levels are influenced by carbohydrate intake and insulin secretion, so VLCDs have been widely proposed as a valid alternative to hypocaloric diets, in terms of improving the outcomes in women with PCOS [210].

A randomized-controlled trial demonstrated that the KD significantly reduces anthropometric parameters and body composition. A VLCD, with an adequate supply of protein intake, preserves the lean body mass. In addition, the observation of a significant reduction in the liver function markers emphasizes VLCD as a treatment of fatty liver, compared to the traditional pharmacological treatment [211]. Zhang et al. also demonstrated a significant reduction in glucose and insulin blood levels, with a significant improvement in insulin resistance. An improvement of the lipid profile was observed, with a significant decrease in triglycerides, total cholesterol, and low-density lipoprotein (LDL), and an increase in high-density lipoprotein (HDL). The LH/FSH ratio, LH total, free testosterone, and dehydroepiandrosterone sulfate (DHEAs) blood levels, were also significantly reduced [212].

An important limitation is the high dropout rate among women with low evidence of a long-term effect, which led to the consideration of VLCDs as safe for short dietary cycles. It could be suitable to shift to a Mediterranean dietary pattern, with physical activity, to achieve results in the long term [213].

### 7.3. Ketogenic Diet on Cardiovascular Risk and Dyslipidemia

Regarding the possible role of VLCKDs on cardiovascular risk, markers are beginning to emerge. In a clinical trial, conducted on 30 adults with metabolic syndrome (MetS) diagnosis and prediabetes or diabetes, and BMI > 25 kg/m^2^, compared the effects of the ketogenic diet (KD), standard American diet (SAD), and standard American diet plus exercise, on health outcomes. The results showed that the KD group had a decrease in weight, body fat, BMI, HA1c, triglycerides, and a higher resting metabolic rate (RMR), after 10 weeks [214]. One study compared a low-carb diet (<30 g/day) to low-fat diet in obese adults. After 6 months, the results showed a drastic decrease in TG for the low-carb diet group, but no significant difference for the total cholesterol (TC), HDL, or LDL [215]. In a study of overweight patients who were followed for two years, divided into groups according to diet (Mediterranean diet, low-fat diet, or low-carb diet), a significant decrease in triglycerides, but also in TG/HDL ratio, was found in the low-carb diet group, and there was an increase in the HDL levels in all the groups. In the low-fat diet group, the decrease in TG/HDL ratio was 12%, while in the low-carb diet group it was 20% [195]. Choi et al. found an improvement in the blood lipid profile in the KD group of obese adults [216]. Lastly, in a 6-month study of obese patients, the KD and low-calorie diet were compared, and showed a decrease in TG, total cholesterol, and LDL, and an increase in HDL [217]. In conclusion, we can say that there are many preliminary studies showing the important effects that the ketogenic diet could have on CVD outcomes. Although a recommendation, different from the current, on fat intake, to prevent the onset of cardiovascular disease, has not yet been firmly established, a good attempt would be to establish a dietary pattern that was able to reduce the increasing incidence of diabetes and obesity, which are both linked to cardiovascular risk.

### 7.4. Ketogenic Diet Metabolic Impact in Children and Adolescents

Recent evidence suggests that more intensive dietary approaches may have benefits, especially for adults with severe obesity and obesity with comorbidities. Based on the efficacy of these approaches in adults, very-low- and low-carbohydrate approaches have been suggested to be beneficial for young people with prediabetes, insulin resistance, or nonalcoholic fatty liver disease [218,219]. Several studies have suggested a possible role for ketogenic diets in obesity in children, but the effects on metabolic parameters in children have been incompletely assessed. Partsalaki et al. compared the efficacy and metabolic impact of ketogenic and low-calorie diets in obese children and adolescents, and showed that the KD produced a greater improvement in weight loss and metabolic parameters than the low-calorie diet. An improvement was observed in the adiponectin concentrations, lipid profile, and metabolic parameters related to insulin sensitivity and resistance in obese children and adolescents [220]. Krebs et al. [221], in an rct on 33 obese adolescents (aged 14.2 ± 0.4) divided in two groups (group 1 high-protein, low-carb (<20 g/day) diet and group 2 low-fat diet). A significant reduction in (BMI-Z) was achieved in both the groups during the intervention, and was significantly greater for the high-protein, low-carb diet group. Both the groups maintained significant BMI-Z reduction at the follow-up. Although no adverse effects were observed in the metabolic profile or cardiac function, a loss of lean mass was also observed in group 1. In another clinical trial, on six obese adolescents aged 12 to 15 years, with a BMI average of 50.9 kg/m^2^ (39.8–63.0 kg/m^2^), following a ketogenic diet for 8 weeks (650 to 725 kcal/day), the authors concluded that a ketogenic diet can be used effectively for rapid weight loss in adolescents with morbid obesity. The loss in lean body mass is blunted, blood chemistries remain normal, and sleep abnormalities significantly decrease with weight loss [222]. Another randomized, controlled 12-week trial, on 30 overweight adolescents, divided into a low-carb diet (LC) group and low-fat diet (LF) group, found an improvement in non-HDL cholesterol levels and greater weight loss in the LC group, and an improvement in LDL cholesterol levels in the LF group. There were no adverse effects on the lipid profiles of the participants in either group. Therefore, this new dietetic frontier may provide additional benefits for young people with obesity, with severe form and with comorbidities [223,224,225]. Lastly, Willi et al. demonstrated rapid weight loss, and less dependence from insulin injections and other antidiabetic drugs, in 20 children (mean age 14 ± 0.4) with T2DM. A reduction in blood pressure has also been demonstrated. In addition to these acute clinical improvements, this treatment option appears to have more lasting benefits on BMI [226].

### 7.5. Ketogenic Diet: Lights and Shadows

Changes in lifestyle (dietary pattern and exercise) are currently recommended as “first-line” therapies in pediatric obesity [5]. However, the poor adherence to this type of treatment raises the question of what other dietary interventions can be effectively applied in children with obesity and metabolic derangement. Although the evidence reported in this review makes it clear that the use of the KD in adult patients with obesity, metabolic syndrome, and T2DM, is now routine, with positive effects in reducing metabolic disorders [187], few studies were found to be conducted on children on this topic in the literature [220,221,222,226]. The reasons limiting this type of study are both the difficulty to apply this type of diet in a child’s daily life (lack of acceptance of certain foods, poor palatability, meals outside the home to be managed by parents), which would lead to scarce compliance in the long term, with consequences on the child’s social sphere, but also the impact of the cost of the diet on the family. Given the selectivity of the diet, another limitation may be the risk of an eating disorder after the restoration of a balanced diet. Moreover, this type of treatment also presents undesirable effects [187], after the first 3–5 days of the regimen, which could lead to a high drop-out rate, even if the appetite-suppressing effect of ketosis could contribute to the success of this diet.

## 8. Conclusions

Pediatric obesity is associated with systemic low-grade inflammation, which has been acknowledged as one of the major drivers of chronic degenerative pathologies, including T2DM and cardiovascular diseases in adult life [1,3,4,5]. The effects of obesity on health, and the costs on health care systems [227], clearly dictate the need to provide nutritional interventions for preventing and treating its complications during childhood. The KD represents a promise therapeutic tool for the treatment of metabolic and cardiovascular risk factors related to obesity in adults. In this review, we explain the hypothesized mechanism of action of KD. The literature also supports a potential role in chronic inflammatory diseases in children [10] and in adolescents who are eligible for bariatric surgery and are affected by PCOS, this treatment is currently proposed. However, clinical studies are needed to evaluate KD as a promising therapeutic tool for the treatment of metabolic and cardiovascular risk factors also in pediatrics.

The direction for future research should be to identify a specific subset of obese children, starting with those aforementioned, who would be less at risk of developing the difficulties described above, which could represent a milestone in clinical studies on the KD in pediatrics.

## Figures and Tables

**Table 1:** Reference values of lipidic pattern in children and adolescents. LDL = low-density lipoprotein; HDL = high-density lipoprotein.

Category	Acceptable	Borderline-High	High
Total cholesterol (mg/dL)	<170	170–199	≥200
LDL cholesterol (mg/dL)	<110	110–129	≥130
Non-HDL cholesterol (mg/dL)	<120	120–144	≥145
HDL cholesterol (mg/dL)	>45	40–45	<40
Triglycerides (mg/dL)			
0–9 years	<75	75–99	≥100
10–19 years	<90	90–129	≥130

**Table 2:** Indications and contra-indications of ketogenic diet in infants.

Indications for Dietary Therapy	Absolute and Relative Contra-Indications
Epilepsy:	Absolute:
- Medically refractory epilepsy, after use of 2 anti-epileptic drugs (AEDs) - West syndrome - Ohtahara syndrome - Febrile infection-related epilepsy syndrome (FIRES) - Severe intolerance to AEDs - Severe myoclonic epilepsy of infancy (Dravet syndrome) - Epilepsy with myoclonic-atonic seizures (Doose syndrome)	- Fatty acid oxidation deficiencies (LCAD, LCHAD, MCAD, OCTN2, CPT1, CPT2) - Pyruvate carboxylase deficiency and other gluconeogenesis defects (fructose 1,6 diphosphatase deficiency) - Glycogen storage diseases (except type 2) - Ketolysis defects - Ketogenesis defects - Porphyria - Prolonged QT syndrome or other cardiac diseases - Liver, kidney or pancreatic insufficiency - Hyperinsulinism
Metabolic and genetic disorders:	Relative:
- Glucose transporter protein 1 (GLUT-1) deficiency - Pyruvate dehydrogenase deficiency (PDHD) - Mitochondrial respiratory chain complex disorders - Angelman syndrome - Tuberous sclerosis complex	- Inability to maintain adequate nutrition - Surgical focus identified by neuroimaging and video-EEG monitoring - Parent or caregiver non-compliance - Growth retardation - Severe gastrointestinal reflux - Familial hypercholesterolemia - Propofol concurrent use

Modified from [130,138]. LCAD = long-chain acyl-CoA dehydrogenase deficiency; LCHAD = long-chain 3-hydroxyacyl-CoA dehydrogenase deficiency; MCAD = medium-chain acyl-CoA dehydrogenase deficiency; OCTN2 = organic cation/carnitine transporter 2; CPT1 = carnitine palmitoyltransferase type 1 deficiency; CPT2 = carnitine palmitoyltransferase type 2 deficiency.

**Table 3:** Indications and contra-indications of ketogenic diet in adults.

Indications	Contra-Indications
- Obesity (BMI > 30 kg/m^2^) - Overweight with co-morbidities (hypertension, non-insulin dependent diabetes mellitus, dyslipidemia, obstructive sleep apnea syndrome, metabolic syndrome, severe osteopathy or arthropathy, NAFLD, PCOS) - Patients eligible for bariatric surgery	- Pregnancy and breastfeeding - Mental and behavioral disorders - Alcohol and other substance abuse - Type 2 diabetes mellitus with significant glucometabolic decompensation and in subjects treated with SGLT2 inhibitors - Severe liver failure (chronic active hepatitis, cirrhosis of the liver) - Renal failure - Acute myocardial infarction within the previous three months, heart failure, unstable angina and arrhythmia - Stroke within the previous three months

Modified from [187]. BMI = body mass index; NAFLD = nonalcoholic fatty liver disease; PCOS = polycystic ovarian syndrome; SGLT2 = sodium–glucose cotransporter 2.

**Table 4:** Blood levels in normal diet, the ketogenic diet and diabetic ketoacidosis.

Blood Levels	Normal Diet	Ketogenic Diet	Diabetic Ketoacidosis
Glucose (mg/dL)	80–120	65–80	>300
Insulin (mU/L)	6–23	6.6–9.4	around 0
Ketone bodies (mM/L)	0.1	7–8	>25
PH	7.4	7.4	<7.3

Modified from [168].

## Data Availability

Not applicable.

## Supplementary Materials

The following are available online at https://www.mdpi.com/article/10.3390/nu13082805/s1, Figure S1: “Nutritional composition of different type of Ketogenic Diet compared to Mediterranean Diet”.
