# Supplementary material for: Metabolic Derangement in Pediatric Patient with Obesity: The Role of Ketogenic Diet as Therapeutic Tool
Source: Nutrients. 2021 Aug 16;13(8):2805. doi: 10.3390/nu13082805 (PMC8400548; doi:10.3390/nu13082805)
Supplement: Supplementary file 1 [file nutrients-13-02805-s001.zip › nutrients-1287348-supplementary.pdf]

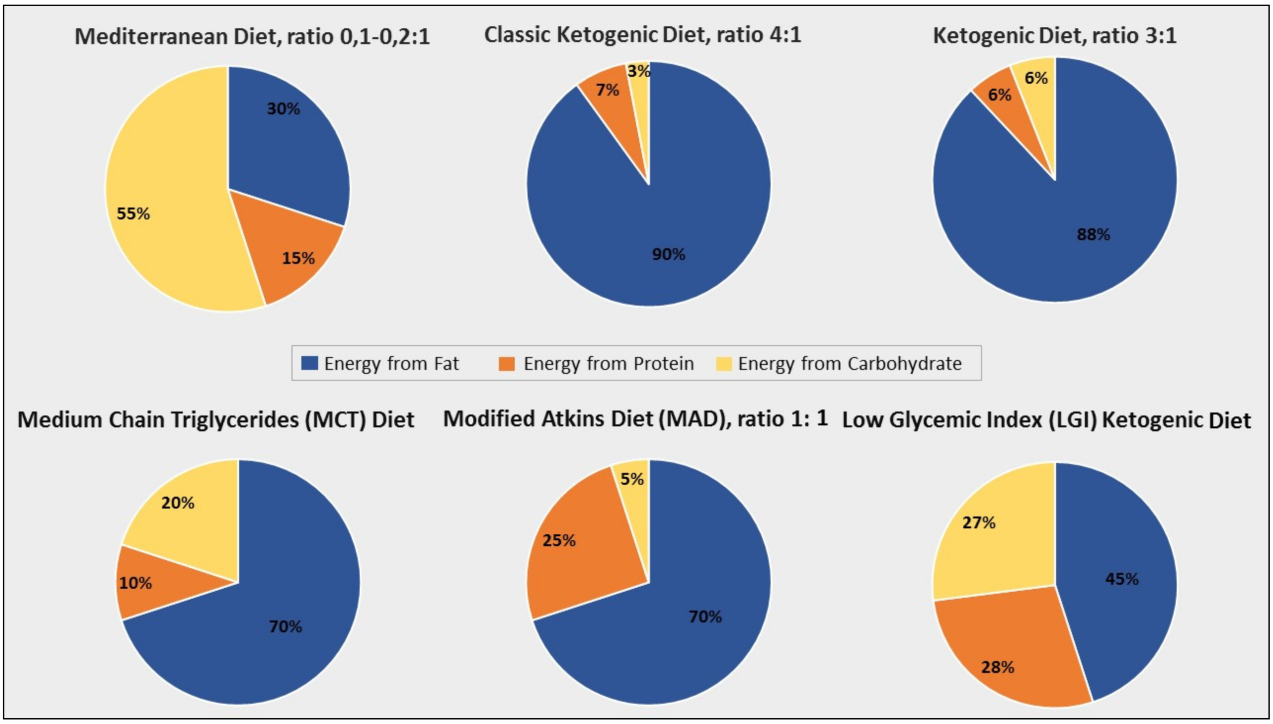

Figure S1 . Nutritional composition of different type of Ketogenic Diet compared to Mediterranean diet
